# Effect of breastfeeding on bone mass from childhood to adulthood: a systematic review of the literature

**DOI:** 10.1186/s13006-015-0056-3

**Published:** 2015-11-20

**Authors:** Ludmila Correa Muniz, Ana Maria Baptista Menezes, Romina Buffarini, Fernando Cesar Wehrmeister, Maria Cecília Formoso Assunção

**Affiliations:** Postgraduate Program in Epidemiology, Federal University of Pelotas, Pelotas, Brazil

**Keywords:** Absorptiometry, Breast feeding, Bone density, Review

## Abstract

**Background:**

Conflicting results exist about the short-and long-term effects of breastfeeding on bone mineral content (BMC) and bone mineral density (BMD). We conducted a systematic review to assess the relationship between method of infant feeding and bone mass in children, adolescents and adults.

**Methods:**

The literature review was concluded in September 2014 in MEDLINE, Web of Science and LILACS databases and articles published between 1998 and 2013 were included. Studies using dual-energy X-ray absorptiometry (DXA) instrument to assess the bone mineral content and/or bone mineral density (BMD) of total body, lumbar spine, femoral neck, or at least one of these sites were included in the review.

**Results:**

From the 648 references identified, eleven were selected, ten of which had a longitudinal design. All studies were conducted in high-income countries, six evaluated the outcome in children, four in adolescents and one in young adults (<35 years). Of the studies that assessed the outcome in childhood, two found a positive association and the others showed a negative effect of being breastfed on bone mass. In adolescence, three studies showed a positive association between being breastfed and bone outcomes. Among adults, a negative effect of being breastfed exclusively for a longer period of time on bone mass was observed only in men. In women, there was no effect of being breastfed on bone mass.

**Conclusions:**

There is no consensus on the effects of method of infant feeding on an individual’s bone mass at different ages.

## Background

Development of the skeleton and subsequent bone health is influenced by a complex interaction of genetic, demographic, socioeconomic, hormonal and environmental factors [1]. Based on evidence from life course studies, early factors inherent to the intrauterine period and early years of life can also influence bone outcomes, as suggested by Barker’s hypothesis [2–4].

To the best of our knowledge, no evidence exists concerning the relationship between intrauterine nutrition and bone mass. However, the association between nutritional exposure during the first year of life and bone mass at different ages has been assessed in some studies. With regard to method of infant feeding, results of investigations into its short and long-term effects on bone mineral content and/or density are conflicting. Some authors have shown a positive effect of being breastfed on bone mass in childhood and adolescence [5–9]. However, other studies have found no association or a negative effect of being breastfed on bone mass outcomes [10–15].

Breastfeeding is universally recognized as the ideal way of feeding infants. The practice is widely recommended by health bodies, given its benefits in terms of nutritional status, cognitive and physical development, reducing mortality for the infant, and the rates of some chronic conditions in adult life [16]. In addition, knowledge on the early modifiable factors influencing bone mass acquisition could allow prevention of osteoporosis and osteoporotic fractures, problems that represent a major social and financial burden for society [17].

Therefore, the objective of the present review was to investigate the relationship between the method of infant feeding and bone mass during childhood, adolescence and/or adult life. In view of the heterogeneity in approaches for assessing the exposure of interest, it was decided to perform a systematic review of the literature as opposed to a meta-analysis.

## Methods

### Search strategy

The literature search, concluded in September 2014, was carried out on the MEDLINE/PubMed, Web of Science (WoS) and Literatura Latino-Americana e do Caribe em Ciências da Saúde (LILACS) databases using free terms for the searching of references. Two groups of key words were created using the connectors “OR” and “AND”, within each group and between the groups, respectively. The terms used for the definition of bone mineral content or density were: “bone mass”, “bone density”, “bone mineral density”, “bone mineral content”, “bone area”, “bone health” and “osteoporosis” (first group). For the exposure of interest, the second group included the terms “breast feeding”, “breast feeding, exclusive”, “breastfeeding”, “breastfeeding, exclusive” and “milk, human”. There were no constraints on publication date or language.

### Inclusion/exclusion criteria of articles

The criteria for inclusion of articles were: being original; having evaluated the relationship between being breastfed and bone mineral content (BMC) and/or bone mineral density (BMD) during childhood, adolescence or in adults; and having used the Dual-energy X-ray Absorptiometry method (DXA) for assessing BMC and/or BMD of the whole body, lumbar spine, neck of the femur or in at least one of these sites.

Studies involving animals or specific population groups such as volunteers, twins and individuals who were sick, premature, with low birth weight or small for gestational age were considered ineligible.

### Selection and quality assessment of articles

After the search process, the references were imported into the software EndNote ×3 (Thompson Reuters, USA) and duplicate references excluded. All the stages of article selection were performed independently by two authors (LCM and RB) and, in cases lacking consensus, a third author (MCA) was consulted. Initially, the authors performed a reading of the titles, excluding those which did not address the association of interest or fulfil the previously defined inclusion criteria. Subsequently, abstracts were read followed by full reading of those articles deemed relevant. After this stage, the lists of references of the selected articles were checked, yielding no further studies.

An adapted version of the scale proposed by Downs and Black was used for the assessment of the methodological quality of the selected articles [18]. Adaptation was required because some items in the original scale did not apply to the cross-sectional, observational or cohort studies. Thus, out of the original 27 items, a total of 17 were assessed as shown in Table 1.

**Table 1:** Criteria for evaluation adapted from Downs and Black [18]

Criteria	
1.	Is the hypothesis/aim/objective of the study clearly described?
2.	Are the main outcomes to be measured clearly described in the Introduction or Methods section?
3.	Are the characteristics of the subjects included in the study clearly described?^a^
4.	Are the interventions of interest clearly described?^b^
5.	Are the distributions of principal confounders in each group of subjects to be compared clearly described?
6.	Are the main findings of the study clearly described?
7.	Does the study provide estimates of the random variability in the data for the main outcomes?
8.	Have all important adverse events that may be a consequence of the intervention been reported?^b^
9.	Have the characteristics of subjects lost to follow-up (refusals) been described?^a^
10.	Have 95 % confidence intervals and/or p values been reported for the main outcomes, except where the p value is less than 0.001?^a^
11.	Were the subjects asked to participate in the study representative of the entire population from which they were recruited?
12.	Were those subjects who were prepared to participate representative of the entire population from which they were recruited?^b^
13.	Were the staff, places, and facilities where the patients were treated, representative of the treatment the majority of patients receive?^b^
14.	Was an attempt made to blind study subjects to the intervention they have received?^b^
15.	Was an attempt made to blind those measuring the main outcomes of the intervention?^b^
16.	If any of the results of the study were based on “data dredging”, was this made clear?
17.	In trials and cohort studies, do the analyses adjust for different lengths of follow-up of patients, or in case-control studies, is the time period between the intervention and outcome the same for cases and controls?^b^
18.	Were the statistical tests used to assess the main outcomes appropriate?
19.	Was compliance with the intervention/s reliable?^b^
20.	Were the main outcome measures used accurate (valid and reliable)?
21.	Were the groups to be compared recruited from the same population?^a^
22.	Were the study subjects recruited over the same period of time?^a^
23.	Were study subjects randomised to intervention groups?^b^
24.	Was the randomised intervention assignment concealed from both patients and health care, Staff until recruitment was complete and irrevocable?^b^
25.	Was there adequate adjustment for confounding in the analysis from which the main findings were drawn?
26.	Were losses of subjects to follow-up taken into account?^a^
27.	Did the study have sufficient power to detect a clinically important effect where the probability value for a difference being due to chance is less than 5 %?

^a^Questions adapted for longitudinal studies

^b^Questions disregarded in this assessment as they did not apply to observational studies

Each item was scored as 0 (no) or 1 (yes), with the exception of item four, which was assigned scores of 0 (no), 1 (partially) or 2 (yes). Final score ranged from zero to 18 points. Based on the score attained, the articles assessed were classified as: low internal validity (0-5 points), intermediate internal validity (6-11 points) and high internal validity (12-18 points). The articles were assessed for these criteria independently by two authors (LCM and RB) and, in cases without consensus, a third author (MCA) was consulted.

## Results

**Fig. 1:**
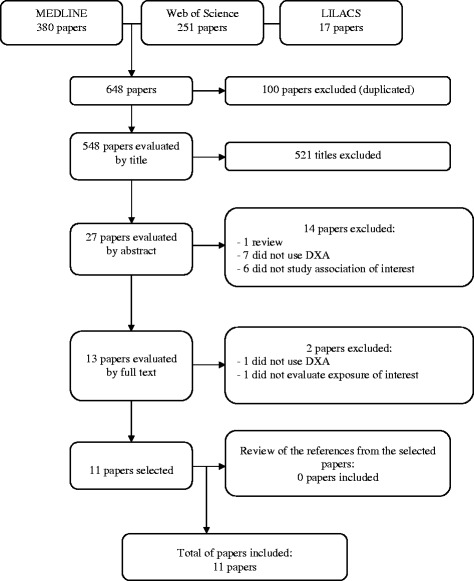
Flow chart of articles assessing the association between method of infant feeding and bone mass measured by DXA at different ages

A total of 648 references were retrieved, comprising 380 on Pubmed, 251 on WoS and 17 on the LILACS databases. After exclusion of 100 to duplicate references, 548 were selected for reading of the titles. Upon conclusion of the selection process, 11 articles were included in the review. All phases of the reference selection process, as well as the number of studies included and excluded at each stage, can be seen in the flow chart depicted in Fig. 1.

The main characteristics of the 11 articles included in this review are given in Table 2. All of the studies were published in English between 1998 [13] and 2013 [5, 8, 11], comprising nine cohort studies [5–10, 12, 14, 15], one randomised trial [11] and one cross-sectional studies [13]. Sample sizes ranged from 35 [13] to 599 [12] subjects. Six of the studies assessed outcome in children (<10 years) [5, 7, 10, 12, 13, 15], four tracked adolescents (10-19 years) [1, 6, 8, 9] and one involved adults (<35 years) [14]. All the studies were carried out in high-income countries. Median score obtained on the Downs & Black assessment was 15 points (minimum 10 and maximum 16 points). Based on the results of this assessment, nine out of the eleven articles were classified as having high internal validity.

**Table 2:** Synthesis of studies that evaluated association between method of infant feeding and bone mineral mass during childhood, adolescence or in adults

Author	Design studies	Age^b^	Sex	N	Exposure	Outcome	Results	Analysis	Adjustment
Place/Year									
Score^a^									
*Papers evaluating children*									
Park [13], Korea 1998, 10^a^	Cross-sectional	2-5 months	22 M, 13 F	35, 18 BF, 17 FF	Exclusive BF (from birth to 5 months)	BMC and BMD spine	No association between breastfeeding and bone mass	Multiple regression	Age and body weight
Butte [10], US 2000, 13^a^	Cohort	0.5, 12 and 24 months	33 M, 43 F	76, 40 BF, 36 FF	Exclusive BF (from birth to 4 months) Duration of BF	BMC whole body	Negative association between breastfeeding and bone mass	Pearson correlation and linear regression	Current weight and length
Jones [7], Australia 2000, 16^a^	Cohort	8 years	215 M, 115 F	330, 175 BF, 151 FF	BF (no, yes) Duration of BF (not, less than 3 months, 3 months or larger)	BMC and BMD whole body, spine, femoral neck	Positive association between breastfeeding and bone mass	Multiple linear regression	Sex, current weight and length, age solids introduced, sports participation, sunlight exposure and current calcium intake
Young [15], EUA 2005, 10^a^	Cohort	4 years	103 M, 75 F	178, 57 BF, 121 FF	Exclusive BF (from birth to 4 months)	BMC and BMD whole body	No association between breastfeeding and bone mass	Analysis of variance	Crude
Harvey [12], UK 2009, 16^a^	Cohort	4 years	318 M, 281 F	599	Duration of BF (never tried, <1 month, 1-3 months, 4-6 months, 7-11 months, 12 months or more)	BMC and BMD whole body	No association between breastfeeding and bone mass	Correlation and linear regression	Bone area, weight, height, childhood milk intake, maternal birth weight, social class, mother’s prudent diet score, parity, physical activity, body build (triceps skinfold thickness) and smoking
Andres [5], EUA 2013, 15^a^	Cohort	3, 6, 9 and 12 months	130 M, 77 F	207	BF (no, yes)	BMC whole body, spine, femoral neck	Positive association between breastfeeding and bone mass	Logistic regression	Age, sex, race, gestational age, birth weight, birth length, food history and socioeconomic status of the mother
*Papers evaluating adolescents*									
Foley [6] Australia 2009, 15^a^	Cohort	16 years	116 M, 67 F	183	BF (no, yes) reported by the mother approximately 1 month after birth	BMC and aBMD whole body and spine	Positive association between breastfeeding and bone mass	Logistic regression	Age, weight and height
Molgaard [9], Denmark 2011, 15^a^	Cohort	17 years	Males, Females	109	Duration of exclusive BF and any BF up to 9 months. If the infants were breastfed at least once a day, they were classified as breastfed	BMC and BMD whole body and spine	Positive association between breastfeeding and bone mass	Correlation	Sex, weight and height
Fewtrell [11], UK 2013, 16^a^	Randomised trial	10 years	193 M, 130 F	323, 120 BF, 203 FF	Exclusive BF (from birth to 12 weeks)	BMC and BMD whole body and spine	No association between breastfeeding and bone mass	Multiple regression	Sex, age, pubertal stage, weight, height, current physical activity and calcium intake
Jones [8] Australia 2013, 16^a^	Cohort	16 years	150 M, 265 F	415	BF at 1 month (no, yes) and BF at 3 months (no, yes), Duration of BF (never, <25 days and ≥ 25 days)	BMD whole body and spine	Positive association between breastfeeding and bone mass	Multivariable linear regression	Sex, age, current weight and height
*Papers evaluating adults*									
Pirilä [14], Finland 2011, 15^a^	Cohort	32 years	76 M, 82 F	158	Duration BF from birth to 12 months (short BF ≤3 months; intermediate BF >3 but <7 months and prolonged BF ≥7 months)	BMC and BMD whole body, spine and femoral neck	Negative association between breastfeeding and bone mass only in men, No association between breastfeeding and bone mass in women	Multivariate analysis of covariance	Gender, dietary intake of calcium, teen-age and current physical activity, smoking history, alcohol consumption, pregnancies, fractures, weight, height, BMI, weight changes during adult life and birth weight

(*n* = 11)

^a^Score by Downs & Black. ^b^ Age outcome. *M* males, *F* females, *BF* breastfeeding, *FF* formula feeding, *aBMD* areal bone mineral density (g/cm^2^)

The method of infant feeding variable was assessed differently by the studies. Three studies [11, 13, 15] assessed the effect of being exclusively breastfed (EBF) compared to being fed infant formula. One study assessed EBF up to the third month of life [11], one from birth to the fourth month [15] and the other up to the fifth month of life [13]. Five studies assessed the effect of being breastfed (yes/no) irrespective of exclusivity or duration [5–8, 10]. Five studies [7–9, 12, 14] assessed the effect of longer duration of being breastfed (irrespective of exclusivity) on bone mass measurements. Only one study [9] assessed the effect of duration of EBF on BMC and BMD.

With regard to bone mass measurements, of the eleven studies, eight assessed BMC and BMD [6, 7, 9, 11–15], two assessed BMC only [5, 10] while one evaluated BMD only [8]. Regarding the anatomical sites studied, three studies assessed bone mass at three sites: whole body, lumbar spine and neck of the femur [5, 7, 14]; three studies assessed whole body only [10, 12, 15]; one investigated lumbar spine only [13] whereas four studies assessed both whole body and lumbar spine [6, 8, 9, 11].

### Effect of being breastfed on bone mineral mass

Among the studies assessing the association of interest in children, three did not find association [12, 13, 15]; two found higher bone mass values among children who were breastfed [5, 7], and one showed that breastfed children had lower whole body BMC compared with those fed with formula, as well as an inverse relationship between duration of total time breastfed and bone mass [10].

The bone mass assessment in adolescents also showed diverse findings. While two studies observed higher bone mass values among those who were breastfed [6, 8]; one reported a direct relation between breastfeeding duration and bone outcomes [9] and one did not find any association [11].

The study evaluating adults found lower BMC and BMO values among men breastfed in infancy for long periods of time. In women, there was no effect of being breastfed on bone mass.

## Discussion

The number of relevant articles found in the present review was small, due to the lack of studies assessing methods of infant feeding compared to bone mineral mass in later life. This low number might be because the present review only assessed the effect of being breastfed on bone mineral mass in individuals born at term with normal weight for gestational age. Studies conducted in preterm newborns were excluded (< 37 weeks’ gestation), since this group potentially has suboptimal mineralization, given that bone mineral deposition is most intense and rapid during the third trimester of pregnancy [19]. Thus, it is likely that effects of breastfeeding on bone mineral mass, over the short and long-term, in preterm infants differs from those observed in term infants.

Although the literature has confirmed the influence of method of infant feeding on body composition in later life, the relationship between being breastfed and bone mineral mass is complex. In this review, the studies that found positive effects of being breastfed (regardless of exclusivity or duration) on BMC and/or BMD showed that formula-fed children had lower bone mass compared to those fed with breast milk [5–8]. Human milk contains lower levels of the nutrients essential for bone mineralization, such as calcium, phosphorus and vitamin D [7], compared to infant formulas and other types of milk. Notwithstanding, there are several explanations for the beneficial effect of breast milk, namely: (1) despite the lower concentration of calcium and phosphorus in human milk, the bioavailability and absorption of these nutrients is greater than other types of milk [7]; (2) consumption of breast milk results in the intake of 200 mg of calcium per day on average [20-22], a sufficient amount to promote good skeletal development during infancy, which can persist into later life; and also, (3) the potentiating effect of breast milk on bone development may stem from a non-nutritional factor [19]. Furthermore, early exposure to breast milk, albeit for a brief period, can lead to changes in the programming of bone cells, resulting in greater bone mass in later life [23].

With respect to breastfeeding duration, Jones et al. showed that children breastfed for longer (≥ 3 months) had higher bone mass values at eight months of life [7]. Molgaard et al. also observed a direct relationship between duration of being breastfed and bone mass in young adults aged 17 years [9]. By contrast, Pirila et al. found that males breastfed for less than three months had higher BMC and BMD at 32 years of age than males breastfed for longer periods (greater than seven months) [14]. One explanation for this finding is that in the first six months of life, the level of calcium present in breast milk is relatively constant, falling by 20-30 % when lactation continues beyond the sixth month [24]. From the sixth month, if the child does not have a nutritionally sufficient and adequate supplementary diet, the calcium level in breast milk may be insufficient to promote normal growth and bone development. In addition, intestinal absorption and urinary excretion of calcium may be positively or negatively affected by other types of milk consumed, impairing the bone mineralization process [25].

It is noteworthy that although all the studies included in this review were conducted in high-income countries, inconsistent associations were found; possibly, due to different contexts regarding breastfeeding between these countries. Studies observing a positive association between being breastfed and bone mass were conducted in countries where the practice of breastfeeding is known to be more commonplace, such as Denmark and Australia [6, 9]. Conversely, in countries where breastfeeding is less widely practiced and promoted, a negative or absence of association was observed [10, 12, 15].

A notable limiting factor of this review was the lack of homogeneity in the approach adopted for assessing breastfeeding among the selected studies. This aspect precluded the determination of an overall measurement of the effect of being breastfed on bone mass from a meta-analysis. Another relevant limitation was that the studies controlled for different confounding factors which, to some degree, hampered comparison of the findings. A negative yet important aspect concerns the low number of studies providing data stratified by gender. Boys and girls are known to reach peak bone mass at different ages, with differences most marked during the prepubertal stage [1, 26, 27].

However, it is important to point out that this review was performed systematically by two authors independently using a broad set of key-words and no limitations on publication date. Of the studies included, most were longitudinal in design, strengthening the causality relationship of the associations found [1, 27]. In addition, the review included studies assessing bone mass using DXA, considered the gold standard for measuring BMC/BMD by the *International Society for Clinical Densitometry* [28]. This inclusion of studies that only employed DXA for assessing bone mass ensured high validity and comparability of the BMC/BMD measurements collected by the studies. Also, the methodological assessment of the articles included in this review suggested they are of sufficient quality, i.e. have high internal validity.

## Conclusions

In conclusion, the results of the present review were conflicting across the different age groups. Analysis of the results stratified by age group revealed a lack of consensus among the studies assessing outcome in infancy; evidence of a positive effect of being breastfed on bone mass measurements in adolescence; and a failure to reach a conclusion regarding the effect in adulthood, given that only one such study was included in the review and that effects were observed only among men. Taken together, these results suggest that being breastfed exerts a greater effect in the long-term, particularly in adolescence, a phase during which around 90 % of peak bone mass is attained [29]. In view of the small number of studies supporting these conclusions, further studies are needed, particularly among low-middle income countries. This information is fundamental**,** not only to inform policies on feeding and nutrition during infancy and adolescence, but also for policies for reducing chronic diseases in adulthood.

## Abbreviations

BMC: bone mineral content
BMD: bone mineral density
DXA: Dual-energy x-ray absorptiometry
EBF: exclusive breastfeeding
LILACS: Literatura Latino-Americana e do Caribe em Ciências da Saúde
WoS: web of science
